# Polygenic Scores of Cardiometabolic Risk Factors in American Indian Adults

**DOI:** 10.1001/jamanetworkopen.2025.0535

**Published:** 2025-03-12

**Authors:** Quan Sun, Jiawen Du, Yihan Tang, Lyle G. Best, Karin Haack, Ying Zhang, Shelley A. Cole, Nora Franceschini

**Affiliations:** 1 Department of Biostatistics, University of North Carolina at Chapel Hill; 2 Now with: Center for Computational and Genomic Medicine, Children's Hospital of Philadelphia, Philadelphia, Pennsylvania; 3 Missouri Breaks Industries Research Inc, Eagle Butte, South Dakota; 4 Texas Biomedical Research Institute, San Antonio; 5 Department of Biostatistics and Epidemiology, University of Oklahoma Health Sciences Center, Oklahoma City; 6 Department of Epidemiology, University of North Carolina at Chapel Hill

## Abstract

**Importance:**

Numerous efforts have been made to include diverse populations in genetic studies, but American Indian populations are still severely underrepresented. Polygenic scores derived from genetic data have been proposed in clinical care, but how polygenic scores perform in American Indian individuals and whether they can predict disease risk in this population remains unknown.

**Objective:**

To study the performance of polygenic scores for cardiometabolic risk factors of lipid traits and C-reactive protein in American Indian adults and to determine whether such scores are helpful in clinical prediction for cardiometabolic diseases.

**Design, Setting, and Participants:**

The Strong Heart Study (SHS) is a large American Indian cohort recruited from 1989 to 1991, with ongoing follow-up (phase VII). In this genetic association study, data from SHS American Indian participants were used in addition to data from 2 large-scale, external, ancestry-mismatched genome-wide association studies (GWASs; 450 865 individuals from a European GWAS and 33 096 individuals from a multi-ancestry GWAS) and 1 small-scale internal ancestry-matched American Indian GWAS (2000 individuals). Analyses were conducted from February 2023 to August 2024.

**Exposure:**

Genetic risk score for cardiometabolic disease risk factors from 6 traits including 5 lipids (apolipoprotein A, apolipoprotein B, high-density lipoprotein cholesterol, low-density lipoprotein cholesterol, and triglycerides), and an inflammatory biomarker (C-reactive protein [CRP]).

**Main Outcomes and Measures:**

Data from SHS participants and the 2 GWASs were used to construct 8 polygenic scores. The association of polygenic scores with cardiometabolic disease was assessed using 2-sided z tests and 1-sided likelihood ratio tests.

**Results:**

In the 3157 SHS participants (mean [SD] age, 56.44 [8.12] years; 1845 female [58.4%]), a large European-based polygenic score had the most robust performance (mean [SD] *R*^2^ = 5.0% [1.7%]), but adding a small-scale ancestry-matched GWAS using American Indian data helped improve polygenic score prediction for 5 of 6 traits (all but CRP; mean [SD] *R*^2^, 7.6% [3.2%]). Lipid polygenic scores developed in American Indian individuals improved prediction of diabetes compared with baseline clinical risk factors (area under the curve for absolute improvement, 0.86%; 95% CI, 0.78%-0.93%; likelihood ratio test *P* = 3.8 × 10^−3^).

**Conclusions and Relevance:**

In this genetic association study of lipids and CRP among American Indian individuals, polygenic scores of lipid traits were found to improve prediction of diabetes when added to clinical risk factors, although the magnitude of improvement was small. The transferability of polygenic scores derived from other populations is still a concern, with implications for the advancement of precision medicine and the potential of perpetuating health disparities, particularly in this underrepresented population.

## Key Points

**Question:**

What is a proper strategy to construct polygenic scores (PGSs) for cardiometabolic risk factors in American Indian adults, and are the scores associated with cardiometabolic disorders?

**Findings:**

In this genetic association study that genotyped 3157 American Indian adults from the Strong Heart Study, performed genome-wide association study (GWAS) analysis, and constructed PGSs for cardiometabolic risk factors, a small-scale ancestry-matched American Indian GWAS was found to improve PGS prediction in this population, and such scores were associated with diabetes.

**Meaning:**

These findings emphasize the importance of including American Indian individuals in genetic studies to avoid bias in precision medicine for implementation of PGS in clinical care.

## Teaser

This genetic association study analyzes the performance of polygenic scores for cardiometabolic risk factors in American Indian adults and their utility in clinical prediction for cardiometabolic disease.

## Introduction

Polygenic scores (PGSs) have demonstrated clinical utility in stratifying high-risk individuals, and they have potential applications in clinical trial design and public health.^1^ However, PGSs have shown bias in performance and tend to benefit individuals of European ancestry disproportionately,^2^ partly due to limited representation of other populations in genome-wide association studies (GWASs). To mitigate the health disparities PGSs may cause in clinical applications, there have been calls for more genetic studies on non-European populations.^2,3,4,5,6^ However, most multi-ancestry genetic studies have limited American Indian participants. For example, only 574 American Indian individuals were included in the non-European GWASs from the Population Architecture Using Genomics and Epidemiology (PAGE) study for C-reactive protein (CRP; 28 537 participants),^3^ and no American Indian individuals were included in the latest multi-ancestry GWAS of lipids that included 1.65 million total individuals and 350 000 non-European participants.^7^ Few studies have considered how PGSs developed in other populations perform in American Indian populations, demonstrating clear underrepresentation^8^; this may exacerbate bias and health inequity in implementing genetic data in clinical care and public health for applications such as disease risk prediction and stratification, diagnosis, and prognosis.

In this study, we address important questions regarding PGS performance in American Indian adults for cardiometabolic risk factors associated with atherosclerosis and inflammation and investigate if such scores are associated with clinical outcomes in this population. We genotyped American Indian adults from the Strong Heart Study (SHS) to advance genetic studies of this population. We performed GWAS for 6 traits, including 5 lipids (apolipoprotein A [APOA], apolipoprotein B [APOB], high-density lipoprotein [HDL] cholesterol, low-density lipoprotein [LDL] cholesterol, and triglycerides [TG]), and an inflammatory biomarker (CRP). We then developed and evaluated the performance of different PGSs in this population, leveraging GWAS data from the UK Biobank (UKB) and the PAGE study. We hypothesized that an ancestry-matched internal cohort would improve PGS performance in this population.

## Methods

### Study Population

This genetic association study used data from the SHS, a large, well-characterized cohort of 4549 self-identified American Indian adults aged 45 years or older, who were recruited from tribal communities in Arizona, Oklahoma, North Dakota, and South Dakota (1989-1991).^9^ The SHS cohort has completed 3 clinical examinations (phase I, 1989-1991; phase II, 1993-1995; and phase III, 1998-1999) and has collected comprehensive data on clinical, lifestyle, and physical measures, as well as medications at each clinical visit, using standardized protocols. Fasting blood and urine samples were obtained at each visit. The SHS protocols were approved by the Indian Health Services institutional review board, the institutional review boards of all participating institutions, and by the participating tribal review boards. This study was considered exempt by the University of North Carolina at Chapel Hilll institutional review board because it is a secondary analysis of the data using deidentified datasets. Participants were selected for genotyping based on having provided written informed consent for genetic research. This study followed the Strengthening the Reporting of Genetic Association Studies (STREGA) reporting guideline.

### Genome-Wide Genotyping and Quality Control

Genome-wide genotyping was performed using the infinium multi-ethnic global-8 array for 1 748 250 markers. After sample and variant quality control, 1 726 345 variants remained for imputation (eMethods in Supplement 1). We performed imputation following our previous procedures^10,11,12^ using TOPMed imputation server. After postimputation quality control, we had 13 210 147 variants for analyses (eMethods in Supplement 1).

### Genome-Wide Association Study

GWAS in SHS was performed for the 6 traits (5 lipids and CRP) using the efficient mixed-model association expedited test^13^ implemented in the Efficient and Parallelizable Association Container Toolbox version 3.3.0 (Center for Statistical Genetics). CRP, HDL and TG were log-transformed. We performed inverse normal transformation of the residuals after adjusting for age, sex, recruitment center, and top 10 genotype principal components (eMethods in Supplement 1).

We used GWAS summary statistics of lipid traits from European UKB (450 865 individuals) and multi-ancestry studies (UKB and PAGE; 33 096 individuals) to derive PGSs (eTable 1 in Supplement 2). The UKB GWAS has been published.^4,5^ We also used non-European UKB GWAS for APOA and APOB biomarkers, given their GWAS were not available from the PAGE study (eMethods in Supplement 1). For other traits, we obtained multi-ancestry GWAS from the PAGE study^3^ for PGS construction.

### PGS Construction

We constructed 8 PGSs and tested these PGSs in 2 samples of SHS individuals (all included SHS participants and an SHS-testing sample) (Table 1). These PGSs were based on 3 external GWAS, 2 linkage disequilibrium (LD) reference panels from the 1000 Genomes Project,^14^ and 2 polygenic risk score (PRS) methods: PRS continuous shrinkage (PRS-CS)^15^ for single-GWAS PGSs and and its cross-population extension (PRS-CSx)^16^ for multi-GWAS PGSs (Table 1, eTable 2 in Supplement 2, and the eMethods in Supplement 1).

**Table 1.** PGS Construction Workflow^**a**^

Genome-wide association study	Linkage disequilibrium	Method	PGS Formula No.
European UKB^b^	European	PRS-CS	1
PAGE or UKB multi-ancestry^b^	European	PRS-CS	2
PAGE or UKB multi-ancestry^b^	Central American	PRS-CS	3
European UKB and PAGE or UKB multi-ancestry^b^	European and Central American	PRS-CSx	4
SHS training^c^	European	PRS-CS	5
SHS training^c^	Central American	PRS-CSx	6
European UKB and SHS training^c^	European and Central American	PRS-CSx	7
European UKB and PAGE or UKB multi-ancestry^c^	European and Central American	PRS-CSx	8

Abbreviations: PAGE, Population Architecture Using Genomics and Epidemiology study; PGS, polygenic score; PRS-CS, polygenic risk score continuous shrinking; PRS-CSx, cross-population polygenic risk score continuous shrinking; SHS, Strong Heart Study; UKB, UK Biobank.

^a^ Eight PGS models were constructed based on different genome-wide association study summary statistics, linkage disequilibrium reference panels, and 2 Bayesian methods: PRS-CS and PRS-CSx. There were 450 865 individuals in the UK Biobank, 33 096 individuals in the PAGE or UKB multi-ancestry bank, and 2000 individuals in the SHS training cohort.

^b^ The PGS was applied to all SHS participants (3157 participants).

^c^ The PGS was applied to SHS testing participants (1157 participants).

We first constructed PGSs using all included SHS participants, aiming to investigate the best combination of GWAS and LD for European or multi-ancestry PGSs in SHS. These PGSs included 3 single-GWAS PGS formulas and a multi-GWAS PGS formula. Specifically, the European UKB formula (formula 1) included UKB European GWAS with European LD. European multi-ancestry (formula 2) included multi-ancestry GWAS with European LD. Central American multi-ancestry (formula 3) included multi-ancestry GWAS with Central American LD, and the UKB multi-ancestry (formula 4) included UKB European GWAS with European LD and multi-ancestry GWAS with Central American LD combined (eMethods in Supplement 1).

To evaluate whether a small ancestry-matched American Indian GWAS could help improve PGS performance, we split the total SHS individuals into 2 groups (1 training group and 1 testing group), assigning all related individuals in training to avoid relatedness across training and testing samples. We performed GWAS on the training group, following the same GWAS protocol, and constructed an additional 4 PGS formulas (Table 1). These included European SHS (formula 5), an SHS GWAS training set with European LD (LD mismatch); Central American SHS (formula 6), an SHS GWAS training set with Central American LD (LD match); UKB and SHS (formula 7), a multi-GWAS PGS using European GWAS with European LD and an SHS GWAS training set with Central American LD; and multi-ancestry and SHS (formula 8), a multi-GWAS PGS using multi-ancestry GWAS with European LD and an SHS GWAS training set with Central American LD (eMethods in Supplement 1).

For all single-GWAS PGSs, we ran PRS-CS-auto with default parameters, because prior studies show that the performance varies little with different tuning parameters.^4,15^ For all multi-GWAS PGSs, we ran PRS-CSx with default parameters and adopted a 2-fold cross-validation strategy to combine the 2 GWAS-specific PGSs following a previous publication^4^ (eMethods in Supplement 1). All the PGS evaluation was based on partial *R*^2^ (eMethods in Supplement 1).

### Association of PGSs With Cardiometabolic Diseases

To assess the clinical prediction of the derived PGSs, we examined the association of lipid PGSs with 5 clinical end points (incident stroke, incident heart failure, incident coronary heart disease, prevalent hypertension, and prevalent diabetes) in the SHS testing sample using the UKB and SHS (formula 7) PGSs. We did not test CRP because the samples were small given that the assay was obtained in the SHS phase II. We first investigated the added value of each single PGS to the clinical end points accounting for baseline covariates (age, sex, and recruitment centers) and clinical risk factors as previously derived specifically for SHS,^17^ including blood pressure, body mass index (calculated as weight in kilograms divided by height in meters squared), LDL, HDL, diabetes status, albuminuria, and cigarette smoking status. We did not include LDL and HDL levels because our PGSs were developed to predict them, and we dropped blood pressure for the hypertension analysis and diabetes status for the diabetes analysis. For each pair of lipid PGS and disease outcome, we fitted a logistic regression model including PGS and other risk factors, and performed a 2-sided *z* test for the lipid PGS.

We additionally evaluated the 5 lipid PGSs jointly (APOA, APOB, HDL, LDL, and TG) considering the following 4 different models: (1) a baseline model including only age, sex, and recruitment centers; (2) a model including baseline covariates and 5 lipid PGSs; (3) a model with baseline covariates and clinical risk factors as described previously; and (4) a model including all the covariates in model 3 as well as the 5 lipids PGSs. We did not include diabetes for prediction of diabetes, and did not include blood pressure for prediction of hypertension, because they defined the corresponding disease. Moreover, HDL and LDL levels were also included in models 3 and 4 as clinical risk factors. We fitted these models using logistic regression and performed a likelihood ratio test with 5 degrees of freedom to test whether our lipid PGSs provide additional value to disease prediction beyond these traditional risk factors.

### Statistical Analysis

Statistical analysis was performed from February 2023 to August 2024. In addition to genetic association tests, we performed 2-sided *z* tests for single PGS associations with clinical outcomes, and 1-sided likelihood ratio tests with 5 degrees of freedom for joint lipid PGSs and cardiometabolic disease models compared with models with only clinical risk factors. The threshold for significance was *P* < .05. All analyses were performed in R version 4.1.0 (R Project for Statistical Computing).

## Results

### Characteristics of Study Participants

The study included 3157 individuals (1845 female [58.4%]) and 13 210 147 postimputation variants. The mean (SD) age at enrollment was 56.44 (8.12) years, ranging from 44.50 to 75.40 years. Most participants were recruited from Oklahoma and South Dakota sites (Table 2). The prevalence of cardiometabolic diseases was 1167 (37.2%) for hypertension, 1213 (39.4%) for diabetes, 360 (11.5%) for incident stroke, 1177 (37.5%) for incident coronary heart disease, and 632 (20.1%) for incident heart failure.

**Table 2.** Characteristics of Strong Heart Study Participants

Variable	Participants, No. (%) (N = 3157)
Age, y	
Mean (SD)	56.44 (8.12)
Median (range)	55.30 (44.50-75.40)
Sex	
Female	1845 (58.4)
Male	1312 (41.6)
Body mass index^a^	
Mean (SD)	30.45 (5.99)
Median (range)	29.72 (15.40-72.36)
Missing	8 (0.25)
Center	
Arizona	428 (13.5)
Oklahoma	1385 (43.9)
South Dakota	1344 (42.6)
Lipid values, mean (SD), mg/dL	
Apolipoprotein A	1.50 (0.32)
Apolipoprotein B	1.12 (0.33)
High-density lipoprotein	46.16 (13.85)
Low density lipoprotein	121.74 (32.20)
Triglycerides	150.51 (150.10)
C-reactive protein, mean (SD), mg/dL	0.68 (1.04)

SI conversion: To convert apolipoprotein a and apolipoprotein to grams per liter, multiply by 0.01; to convert high- and low-density lipoportein to millimoles per liter, multiply by 0.0259; to convert triglycerides to millimoles per liter, multiply by 0.0113; to convert C-reactive protein to milligrams per liter, multiply by 10.

^a^ Body mass index was calculated as weight in kilograms divided by height in meters squared.

### GWAS of Lipid and Inflammatory Traits

The SHS GWAS (3157 participants) replicated some known genome-wide significant loci, including the *ZPR1* locus on chromosome 11 for APOA, HDL, and TG; the chromosome 16 *CETP* locus for HDL; the chromosome 1 *CELSR2* locus and the chromosome 11 *APOE* locus for LDL; and the *HNF1A* locus on chromosome 12 for CRP (eFigures 1-6 in Supplement 2). After conditioning on known variants, no variant associations remained, indicating that prior findings accounted for associations in these regions.

### Performance of PGS in American Indian SHS Participants

We first focused on testing PGSs in all 3157 SHS participants and calculated 4 PGSs with formulas 1 (European UKB), 2 (European multi-ancestry), 3 (Central American multi-ancestry), and 4 (UKB and multi-ancestry) (Table 1, the eMethods in Supplement 1, and eTable 2 in Supplement 2). We found that for all traits but APOA and TG, the PGS for formula 4 achieved the highest or close to the highest *R*^2^ (eFigure 7 in Supplement 2 and eTable 3 in Supplement 1), supporting the importance of including multi-ancestry GWAS in PGS to capture the genetic risk in this population. The PGS based on PAGE multi-ancestry GWAS and Central American LD (formula 3; mean [SD] *R*^2^, 6.0% [2.4%]), which had a 13 times smaller sample size than UKB, slightly outperformed the PGS with formula 1 (*R*^2^, 5.5%) for LDL. However, there was almost no predictive power using UKB non-European GWAS for APOA and APOB (PAGE GWAS not available), likely due to differences in population genetic structure between non-European individuals in the UK vs the US. We note that the largest number of PAGE participants were Hispanic and Latino individuals who have varying proportions of Central American and European ancestry admixture and have been shown to achieve satisfactory performance with PGS based on European LD.^8,18^ These results indicate that American Indian individuals, like Hispanic and Latino individuals, can benefit from combining large European and multi-ancestry GWAS for deriving PGSs, while the multi-ancestry GWAS alone may not represent the genetic variation in American Indian individuals well and should be applied cautiously.

### PGS Including a Small Ancestry-Matched GWAS in American Indian Individuals

We then attempted to investigate whether adding a small sample GWAS of American Indian adults with matched ancestry could improve PGS performance in this population. We split the total SHS individuals into 2000 for training and the remaining 1157 individuals for testing and constructed an additional 4 PGS formulas, including formulas 5 (European SHS), 6 (Central American SHS), 7 (UKB and SHS), and 8 (multiple-ancestry and SHS)(Table 1 and the eMethods in Supplement 1). The following results are for the SHS testing set (1157 individuals).

**Figure 1.**
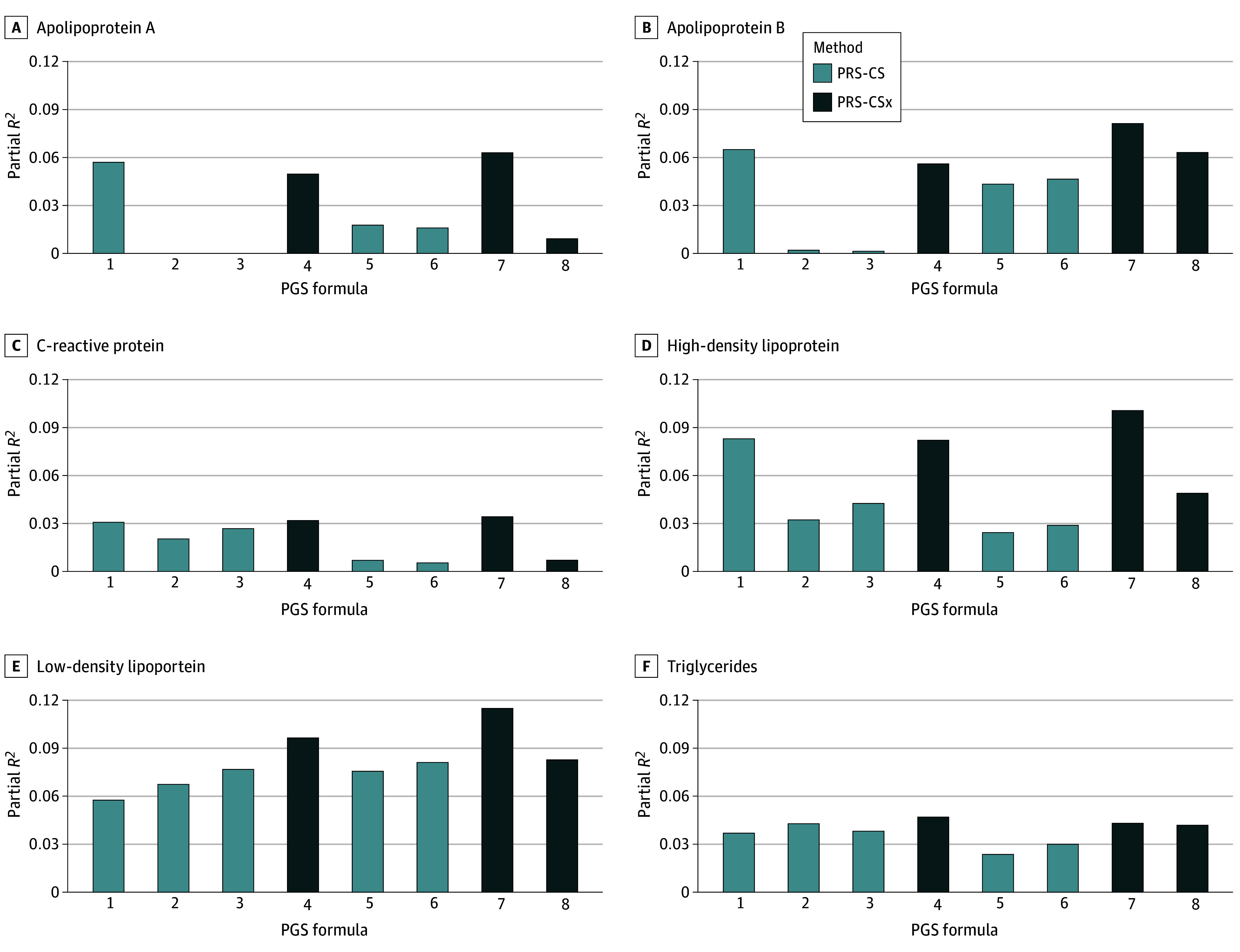
Polygenic Score (PGS) Results in Strong Heart Study (SHS) Participants PGS performance in 1157 SHS testing individuals. Eight PGS formulas for all SHS participants were constructed, including 5 single-genome-wide association study (GWAS) PGSs (European UK Biobank [1], European multi-ancestry [2], Central American multi-ancestry [3], European SHS [5], and Central American SHS [6]) using polygenic risk score continuous shrinkage (PRS-CS) and 3 multi-GWAS PGSs (UKB and multi-ancestry [4], UKB and SHS [7], and multi-ancestry and SHS [8]) using a cross-population extension (PRS-CSx).

We found that the PGS for formula 1 (European UKB) achieved the most robust performance (highest *R*^2^ for 4 of the 6 traits) among all the single-GWAS PGSs (mean [SD] *R*^2^ = 5.0% [1.7%]), likely due to the large GWAS sample size. However, PGSs based on a small SHS training GWAS (formula 5 [European SHS]: *R*^2^, 7.6%; formula 6 [Central American SHS]: *R*^2^, 8.1%) performed better than the formula 1 PGS for LDL (*R*^2^ = 5.8%), although the sample size was 200 times smaller than UKB. They also outperformed PGSs based on multi-ancestry GWAS (formula 2 [European multi-ancestry]: *R*^2^, 6.8%; formula 3 [Central American multi-ancestry]: *R*^2^, 7.7%). In addition, the multi-GWAS PGSs that included UKB European and SHS training GWAS (formula 7) demonstrated the best performance among the 3 multi-GWAS PGSs for 5 of the 6 traits, with a mean (SD) *R*^2^ of 7.6% (3.2%) (Figure 1 and eTable 4 in Supplement 2).

### Association of Lipids PGSs With Cardiometabolic Diseases

To demonstrate the potential clinical utility of PGS in American Indian populations, we conducted association analyses of our best PGSs (formula 7 [UKB and SHS] PGSs) for lipid traits with several clinical end points, including prevalent hypertension, diabetes, incident stroke, heart failure, and coronary heart disease, in the SHS-testing samples (1157 individuals). First, we investigated the value of PGS for each lipid trait in addition to baseline covariates (age, sex, and recruitment centers) and clinical risk factors from disease risk equations derived for American Indian individuals as previously reported,^17^ without HDL and LDL levels (eMethods in Supplement 1). We found 2 significant protective associations for diabetes, specifically for the HDL PGS (odds ratio per standard unit increase of PGS, 0.49; 95% CI, 0.32-0.76; *P* = 1.4 × 10^−3^) and the LDL PGS (odds ratio per standard unit increase of PGS, 0.47; 95% CI, 0.29-0.76; *P*  = 2.3 × 10^−3^) (eTable 5 in Supplement 2).

**Figure 2.**
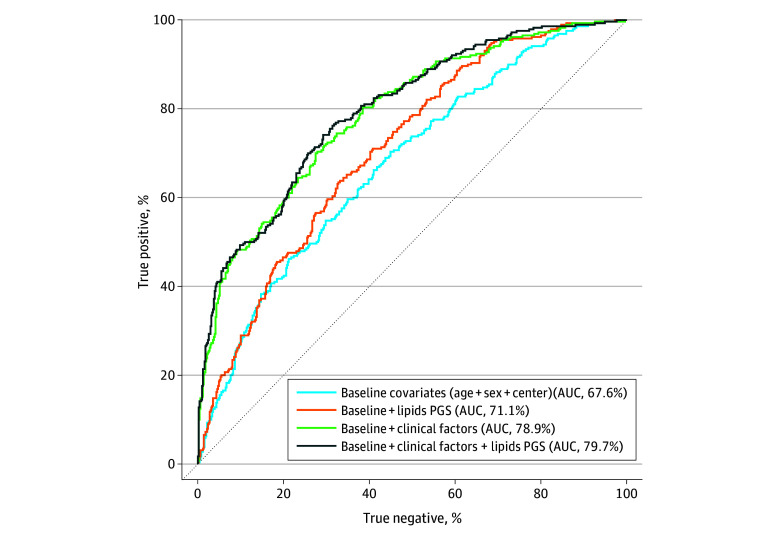
Receiver Operating Characteristic Curve for Diabetes Among 4 Models These models include (1) baseline covariates (age, sex, and recruitment centers), (2) baseline covariates and lipids polygenic scores (PGSs), (3) baseline and traditional clinical risk factors, and (4) baseline, traditional clinical factors, and lipids PGSs. PGSs include UK Biobank and Strong Heart Study for apolipoprotein A, apolipoprotein B, high-density lipoprotein, low-density lipoprotein, and triglycerides. For model 1 vs model 2: likelihood ratio test *P* = 5.6 × 10^−7^. Model 3 vs model 4: likelihood ratio test *P* = 3.8 × 10^−3^. AUC indicates area under the curve.

Second, to evaluate the joint effects of all 5 lipid PGSs, we compared models with these PGSs with a baseline model that only included age, sex, and recruitment centers (eMethods in Supplement 1). We observed that including lipid PGSs improved the prediction of diabetes; area under the curve (AUC) improved from 67.7% (95% CI, 63.8%-71.5%) to 71.7% (95% CI, 67.5%- 74.7%) (*P* = 5.6 × 10^−7^) (Figure 2). To further demonstrate the value of our PGSs in addition to traditional clinical risk factors for cardiometabolic diseases, we compared models with and without lipid PGSs using all the clinical risk factors as described previously^17^ (eMethods in Supplement 1), including HDL and LDL levels. Our results showed that even with all these risk factors considered, including lipid PGSs significantly improved the prediction of diabetes; the AUC improved from 78.9% (95% CI, 75.6%-82.1%) to 79.7% (95% CI, 76.5%-82.9%) (likelihood ratio test *P* = 3.8 × 10^−3^) (Figure 2), although the absolute improvement was small (AUC, 0.86%; 95% CI, 0.78%-0.93). There was no statistically significant improvement in the AUC for other outcomes (eTable 6 in Supplement 2). These results highlight the potential clinical utility of lipid PGSs in risk prediction when added to clinical risk factors in American Indian populations, especially for diabetes. However, the power of PGSs is still limited, making PGS development considering diverse populations a pressing need.

## Discussion

As the sample size in GWAS increases, the importance of including diverse global populations in genetic association studies has been recognized.^3,5,19^ However, studies focusing on the American Indian populations are still lacking, which could amplify health disparities if PGSs are used for screening, diagnosis or prognosis.^2^ Our genetic association study of American Indian individuals from SHS focused on lipid and inflammatory traits, given their relevance to atherosclerosis and cardiometabolic disease, which are highly prevalent in American Indian individuals. We constructed different PGSs and assessed their performance in this population. To our knowledge, this is the first study to develop PGS for lipids in American Indian individuals.

Our main findings suggest that including a small sample of ancestry-matched American Indian individuals can improve PGS performance in this population, although the absolute improvement in *R*^2^ was small. We anticipate larger improvements in prediction when larger samples of American Indian GWAS are available. In addition, differences in allele frequencies between SHS and other populations^20^ (eFigures 8-11 in Supplement 1) support the reason why a small ancestry-matched cohort (UKB and SHS [formula 7]) showed greater improvement than a larger multi-ancestry GWAS that was more than 10 times larger (UKB multi-ancestry [formula 4]) for the PGSs in American Indian individuals. It also highlights that bias in GWAS can perpetuate inequalities for some populations if PGSs are applied to clinical care and public health decisions. This is especially important in the US, given the diversity in the population.

We also demonstrated that lipid PGSs (UKB and SHS [formula 7]) added to the risk prediction of diabetes in this population beyond traditional clinical risk factors. These clinical risk factors included measured HDL and LDL levels, indicating that lipid PGSs were valuable for disease prediction in addition to the actual lipid levels. However, the magnitude of improvement in AUC was small, which may be due to both the small sample size and the underlying genetic architecture of the traits. Although findings were not statistically significant for other outcomes, we argue that PGSs may prove valuable when larger samples of American Indian individuals are used for PGS derivation. Lipid PGSs may capture lifetime disease risk that could be used for primary disease prevention, with some advantages over assessing risk based on measured lipid levels at a single time point. Such scores have shown to have an independent effect beyond the clinical biomarkers that they were derived from as shown in our results for diabetes.

### Limitations

Our study provides important information to guide the derivation of PGSs for American Indian populations. However, there are some limitations warranting future investigations. First, despite being one of the largest cohorts for American Indian individuals, our sample size was still relatively small, limiting the power to detect novel associations. Second, we also observed a certain degree of relatedness in this cohort. Therefore, to balance between unbiased PGS evaluation and the maximum possible sample sizes, we split the total 3157 individuals into 2000 for training and 1157 for testing, rather than performing cross-validation. Third, we did not perform genetic similarity comparisons of SHS participants with 1000 Genomes Project populations, nor any global and local ancestry analyses, due to our agreements with tribes. Fourth, our comprehensive PGS constructions only considered European and multi-ancestry GWAS without integrating GWAS for all possible populations (eg, African or Asian populations). This is, in part, because previous findings suggest that other ancestry GWAS, especially African GWAS, do not contribute to polygenic prediction for American Indian individuals.^8^ In addition, we only considered European and Central American LD references in PGS construction for multi-ancestry GWAS because the largest number of participants in these GWAS were Hispanic or Latino, for whom European and Central American are the 2 most closely relevant populations. Future studies may investigate whether other LD references may improve the results. Furthermore, we did not test CRP PGS in our association with clinical end points because it was measured in a subset of individuals with high missing rates (21.2%) (eTable 1 in Supplement 2).

## Conclusions

This genetic association study found that inclusion of American Indian populations in PGS construction improved PGS performance in this population, although the improvement was small. We found that PGS of lipid traits improves diabetes risk prediction in addition to traditional clinical risk factors in American Indian adults. We anticipate that PGS utility will be improved in American Indian individuals when larger genetic studies in this population are available.

## Notes

Supplement 1.
eMethods.
**eFigure 1.** Manhattan Plot for APOA GWAS in SHS **eFigure 2.** Manhattan Plot for APOB GWAS in SHS **eFigure 3.** Manhattan Plot for CRP GWAS in SHS **eFigure 4.** Manhattan Plot for HDL GWAS in SHS **eFigure 5.** Manhattan Plot for LDL GWAS in SHS **eFigure 6.** Manhattan Plot for TG GWAS in SHS **eFigure 7.** PGS Results in All SHS Participants **eFigure 8.** Smoothscatter Plots Comparing the Allele Frequency Between SHS and European Population in TOP-LD **eFigure 9.** Smoothscatter Plots Comparing the Allele Frequency Between SHS and African Population in TOP-LD **eFigure 10.** Smoothscatter Plots Comparing the Allele Frequency Between SHS and East Asian Population in TOP-LD **eFigure 11.** Smoothscatter Plots Comparing the Allele Frequency Between SHS and South Asian Population in TOP-LD
eReferences.

Supplement 2. **eTable 1.** Sample Size in External GWAS for PGS Construction in SHS **eTable 2.** An Explanation Table for PGS Construction **eTable 3.** PGS Results for All SHS Participants **eTable 4.** PGS Results for SHS Testing Subset **eTable 5.** Pairwise Association Analysis Between Clinical Endpoint and Lipid Trait PGS **eTable 6.** Likelihood Ratio Test (LRT) Results

Supplement 3.
Data Sharing Statement
