# Supplementary material for: Polygenic Scores of Cardiometabolic Risk Factors in American Indian Adults
Source: JAMA Netw Open. 2025 Mar 12;8(3):e250535. doi: 10.1001/jamanetworkopen.2025.0535 (PMC11904716; doi:10.1001/jamanetworkopen.2025.0535)
Supplement: Supplement 1. — eMethods. eFigure 1. Manhattan Plot for APOA GWAS in SHS eFigure 2. Manhattan Plot for APOB GWAS in SHS eFigure 3. Manhattan Plot for CRP GWAS in SHS eFigure 4. Manhattan Plot for HDL GWAS in SHS eFigure 5. Manhattan Plot for LDL GWAS in SHS eFigure 6. Manhattan Plot for TG GWAS in SHS eFigure 7. PGS Results in All SHS Participants eFigure 8. Smoothscatter Plots Comparing the Allele Frequency Between SHS and European Population in TOP-LD eFigure 9. Smoothscatter Plots Comparing the Allele Frequency Between SHS and African Population in TOP-LD eFigure 10. Smoothscatter Plots Comparing the Allele Frequency Between SHS and East Asian Population in TOP-LD eFigure 11. Smoothscatter Plots Comparing the Allele Frequency Between SHS and South Asian Population in TOP-LD eReferences. [file jamanetwopen-e250535-s001.pdf]

Supplemental Online Content

Sun Q, Du J, Tang Y, et al. Polygenic scores of cardiometabolic risk factors in American Indian adults. *JAMA Netw. Open*. 2025;8(3):e250535. doi:10.1001/jamanetworkopen.2025.0535

eMethods
eFigure 1. Manhattan Plot for APOA GWAS in SHS
eFigure 2. Manhattan Plot for APOB GWAS in SHS
eFigure 3. Manhattan Plot for CRP GWAS in SHS
eFigure 4. Manhattan Plot for HDL GWAS in SHS
eFigure 5. Manhattan Plot for LDL GWAS in SHS
eFigure 6. Manhattan Plot for TG GWAS in SHS
eFigure 7. PGS Results in All SHS Participants
eFigure 8. Smoothscatter Plots Comparing the Allele Frequency Between SHS and European Population in TOP-LD
eFigure 9. Smoothscatter Plots Comparing the Allele Frequency Between SHS and African Population in TOP-LD
eFigure 10. Smoothscatter Plots Comparing the Allele Frequency Between SHS and East Asian Population in TOP-LD
eFigure 11. Smoothscatter Plots Comparing the Allele Frequency Between SHS and South Asian Population in TOP-LD
eReferences

This supplemental material has been provided by the authors to give readers additional information about their work.

© 2025 Sun Q et al. *JAMA Network Open*.

eMethods

SHS CRP and lipid phenotypes

CRP was measured in a subset of participants at Phase II using an enzyme-linked immunosorbent assay with purified CRP and anti-CRP antibodies from CalBioChem (La Jolla, California) [1]. This assay has been used in the validation of the commercially available assay for high-sensitivity CRP [2]. The coefficient of variation was approximately 8%. CRP was log-transformed for analysis. Total cholesterol, TG, LDL, HDL (mg/dL), APOA and APOB were measured in fasting blood samples using standard assays and procedures have been published [3]. HDL and TG were log-transformed for analysis.

SHS genotype quality control and imputation

Array genotyping quality control for samples excluded those with a missing rate > 0.05, sex mismatch and duplicates (total n = 64 excluded). Genetic variants were excluded if they did not map to chromosome positions or had more than 0.05 missing genotypes, leaving a total of 1,726,345 variants. We computed the genetic relatedness and principal components (PCs) using PLINK 1.9 [4]. Imputation was performed following our previous procedure [5–7] using the TOPMed freeze 8 reference panel [8] from TOPMed imputation server [9], with Eagle v2.4 [10] for phasing and Minimac4 [11] for imputation. A total of 13,210,147 post-imputation variants with an imputation quality $R^2 \geq 0.3$ and minor allele count (MAC) ≥ 5 were used in analyses.

SHS GWAS

GWAS in SHS (n = 3,157 after exclusions) was performed using the EMMAX test [12] implemented in EPACTS v3.3.0, controlling for population stratification and sample relatedness. Traits were adjusted for age, sex, recruitment center, and top 10 genotype PCs as covariates, and the residuals from the linear regression models were inverse-normal transformed. In the first step of GWAS, we inferred the dense kinship matrix using high-quality variants with MAF > 1% and missing rate < 1%. In the association step, we tested imputed variants with a MAC ≥ 5 and estimated imputation quality $R^2 \geq 0.3$.

UKB GWAS

GWAS of UKB EUR individuals (n = 450,865) was performed using REGENIE [13] due to its computational efficiency for large-scale cohorts. We leveraged the imputed data released from UK Biobank and removed variants with imputation INFO scores < 0.3 and MAC < 20 [14,15]. For APOA and APOB, because their GWAS were not available from the PAGE study, we meta-analyzed UK Biobank GWAS of African, South Asian, and East Asian populations obtained from a published study [14] using the latest version of METAL [16] and used these multi-ancestry GWAS results to derive PGS.

Allele frequency comparison across populations

© 2025 Sun Q et al. *JAMA Network Open*.

We compared the allele frequencies between SHS and four reference populations (Europeans [EUR], Africans [AFR], East Asians [EAS] and South Asians [SAS]) in a reference database, TOP-LD [17], for variants with $p < 1e-4$ in our SHS GWAS.

PRS methods (PRS-CS and PRS-CSx)

PRS-CS [18] is a Bayesian method to construct PGS using GWAS results and LD reference panels, and PRS-CSx [19] is its multi-ancestry extension, which takes multiple GWAS results and LD reference panels from different populations to improve PGS accuracy. For all single-GWAS PGSs, we ran PRS-CS-auto with default parameters, as prior studies show that the performance varies little with different tuning parameters [15,18]. For all multi-GWAS PGSs, we ran PRS-CSx with default parameters, and adopted a two-fold cross-validation strategy to combine the two GWAS-specific PGSs following a previous publication [15] . After calculating the posterior effect size of each variant, we adopted a two-fold cross-validation strategy to combine the two GWAS-specific PGSs [15]. We split the testing dataset into two equally sized samples (A and B), and used sample A for deriving the weights to calculate the linearly combined PGS for each individual in sample B. Then, we derived the weights in sample B and similarly calculated PGS for every individual in sample A.

Rationale of PGS construction

We first constructed PGSs using all SHS participants (n = 3,157) for testing, aiming to investigate the best combination of GWAS (EUR or multi-ancestry) and LD (EUR or AMR) for PGSs in the SHS, leveraging GWAS results from UK Biobank [14,15] and PAGE studies [20] (**Methods**). Since PAGE did not have GWAS for APOA and APOB traits, we used meta-analysis results from published GWAS for the UK Biobank Africans, South Asians and East Asians [14] to represent a multi-ancestry GWAS (n = 21,721), and PAGE GWAS results for other traits (n = 35,000). We refer to these GWAS results as multi-ancestry GWAS (see **Methods**). For EUR GWAS, we used the UKB GWAS results (n = 450,865). The detailed sample sizes for each trait are shown in **eTable 1**. These PGSs included UKB_eur, multi_eur, multi_amr, and UKB+multi (**Methods**). The rationale behind using both EUR and AMR LD references for multi-ancestry GWAS is to compare the choice of reference panels for PGS construction, given that large-scale Hispanic/Latino individuals were included in the multi-ancestry GWAS.

To evaluate whether a small ancestry-matched American Indian GWAS could help improve PGS performance, we constructed PGSs using a subset of SHS American Indian data that we then applied to the testing SHS American Indian set (n = 1,157). We additionally constructed SHS_eur, SHS_amr, UKB+SHS and multi+SHS (**Methods**). The first two are single-GWAS PGSs (SHS_eur and SHS_amr) based on SHS-training (n = 2,000) GWAS, and the latter two (UKB+SHS and multi+SHS) are multi-GWAS PGSs combining external GWAS with small-scale SHS-training (n = 2,000) GWAS.

Note that we used underscores to name single-GWAS PGS to accommodate the different LD reference panels with the same GWAS and adopted a plus sign in the naming for multi-GWAS PGS to emphasize their multi-GWAS feature. We did not consider LD mismatch in a multi-GWAS setting due to its complexity, though arguably no LD reference would be appropriate for multi-ancestry GWAS.

All the PGS evaluation was based on partial R^2, calculated as the squared Pearson correlation between PGS and traits after adjusting for all the covariates, which reflects the trait variance explained by PGS.

© 2025 Sun Q et al. *JAMA Network Open*.

Cardiometabolic disease definitions

Prevalent hypertension was defined by a blood pressure >140/90 mmHg or use of blood pressure-lowering medications (n total = 3,139; n cases = 1,167) at baseline visit. Prevalent diabetes was defined by a fasting glucose >126 mg/dL or A1c≥ 6.5% and/or use of diabetic medications (n total = 3,077; n cases = 1,213) at Phase I (baseline) visit. The high prevalence of diabetes aligns with a recent publication for the age distribution of our study cohort [21]. Incident stroke was defined by fatal and nonfatal total stroke events occurring between the baseline examination and last follow-up among participants without a prior history of stroke (n total = 3,138; n cases = 360). Incident CHD was defined by fatal and nonfatal events (definite myocardial infarction [MI], definite CHD, ECG-evident definite MI, cardiac procedures, including percutaneous transluminal coronary angioplasty or coronary artery bypass graft) occurring between the baseline examination and last follow-up among participants without a history of CHD (n total = 3,138; n cases = 1,177). Incident fatal and nonfatal heart failure (HF) events were ascertained from baseline to last follow-up among participants without a history of cardiovascular disease (n total = 3,138; n cases = 632). We used validated events obtained from surveillance, mortality, and medical records with a follow-up up to December 31st, 2022 [22].

© 2025 Sun Q et al. *JAMA Network Open*.

eFigure 1. Manhattan plot for APOA GWAS in SHS.

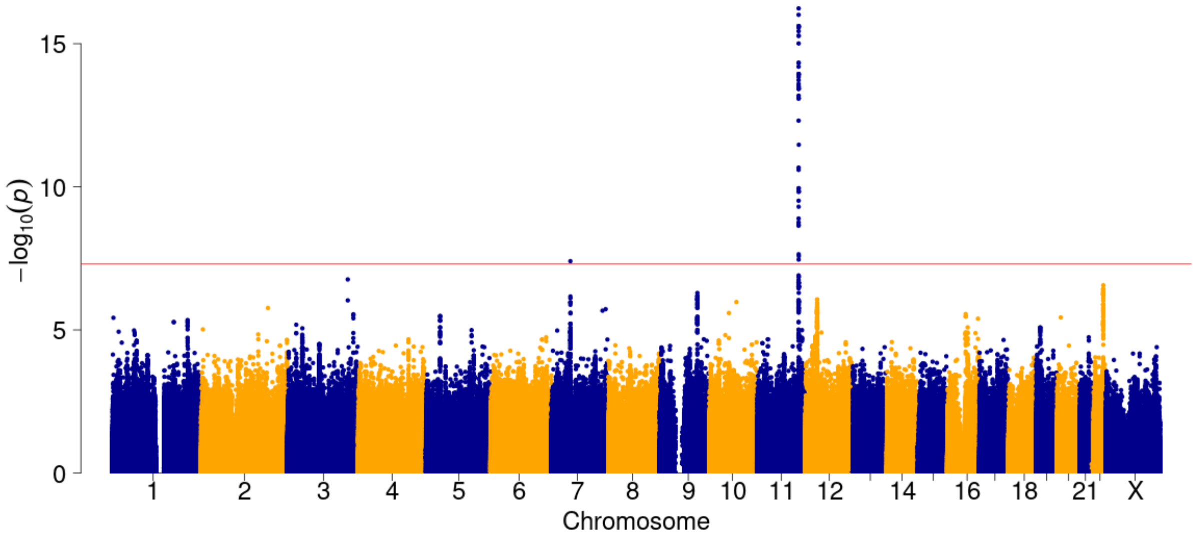

eFigure 2. Manhattan plot for APOB GWAS in SHS.

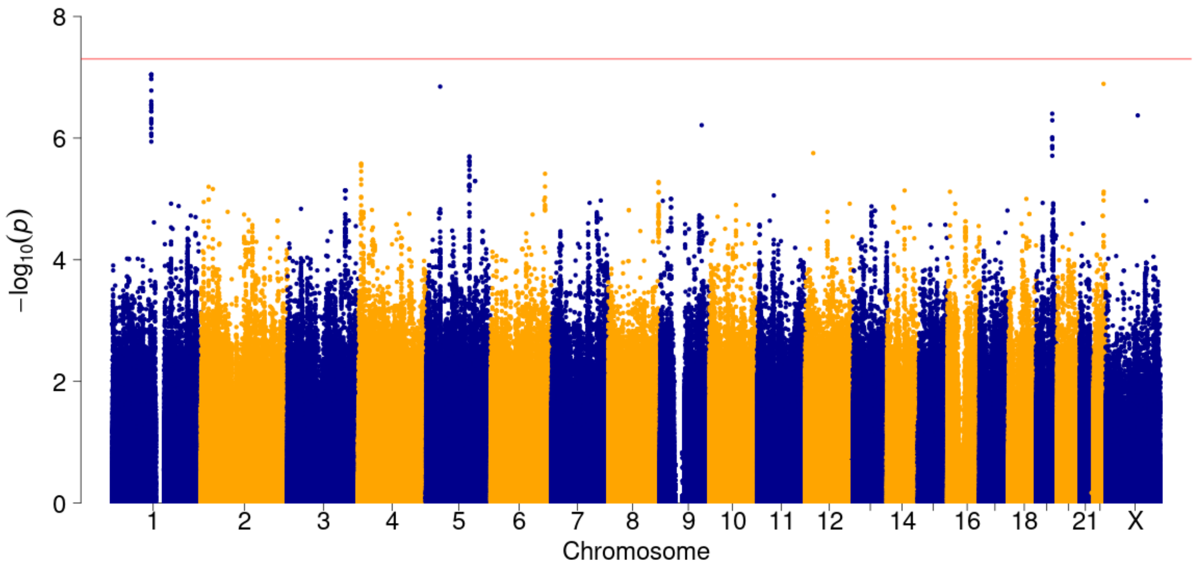

© 2025 Sun Q et al. *JAMA Network Open*.

eFigure 3. Manhattan plot for CRP GWAS in SHS.

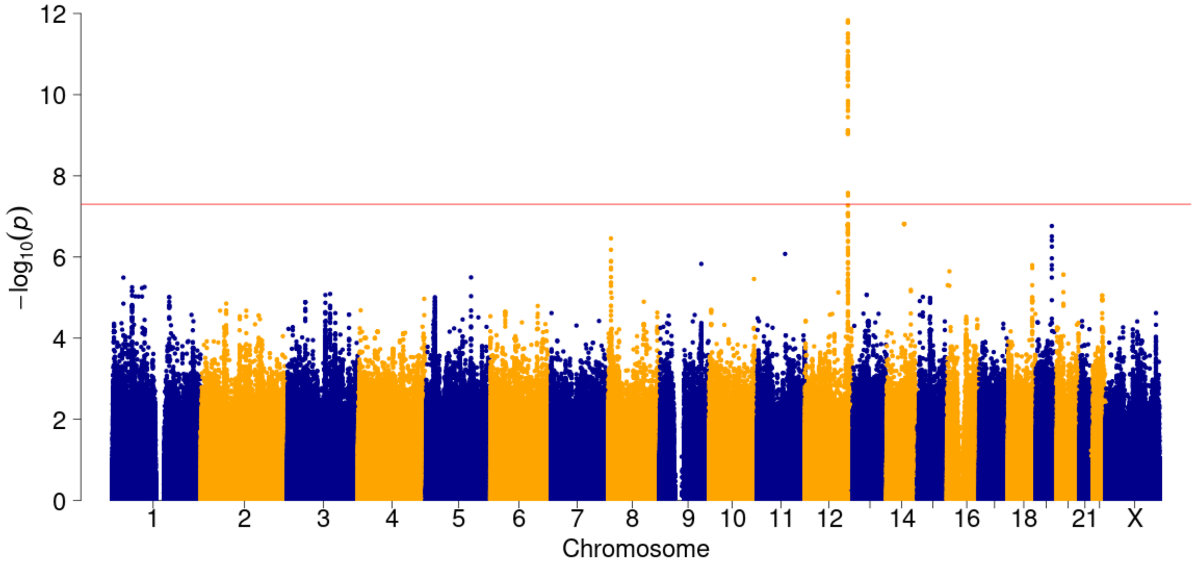

eFigure 4. Manhattan plot for HDL GWAS in SHS.

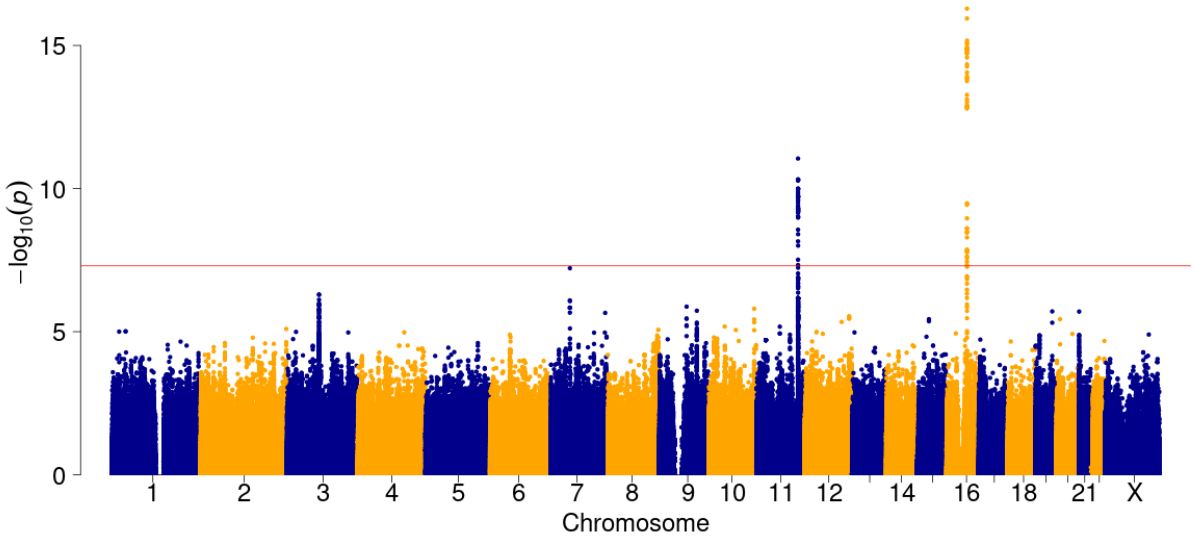

© 2025 Sun Q et al. *JAMA Network Open*.

eFigure 5. Manhattan plot for LDL GWAS in SHS.

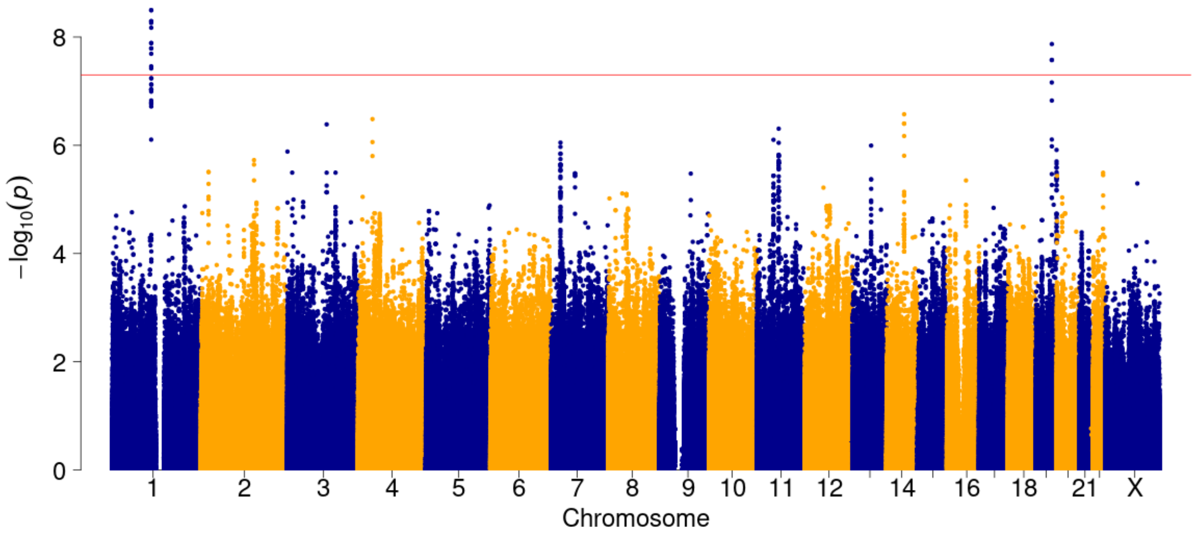

eFigure 6. Manhattan plot for TG GWAS in SHS.

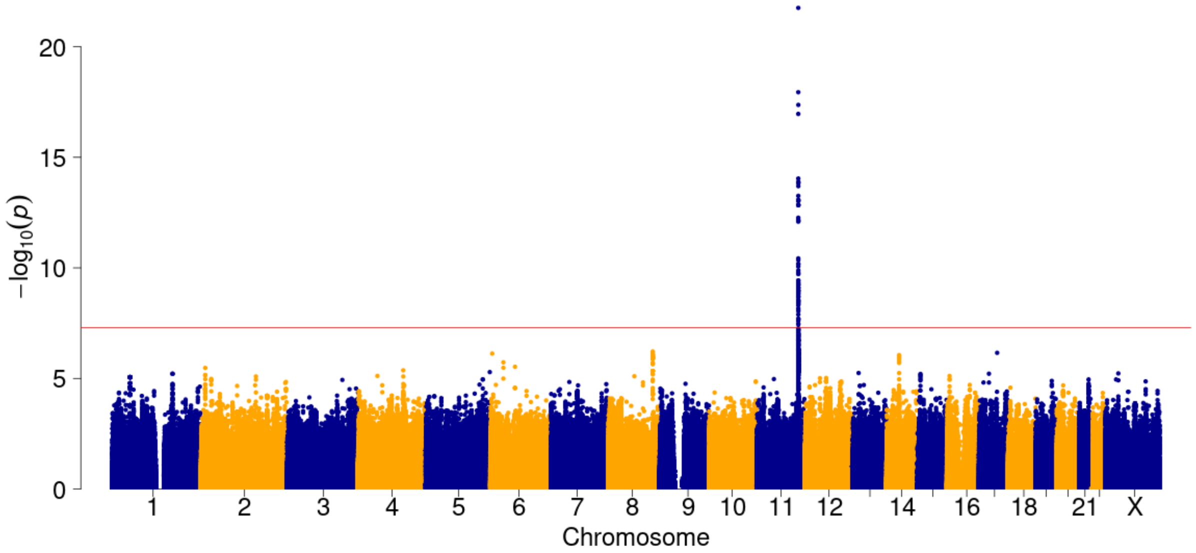

© 2025 Sun Q et al. *JAMA Network Open*.

eFigure 7. PGS results in all SHS participants (n = 3,157).

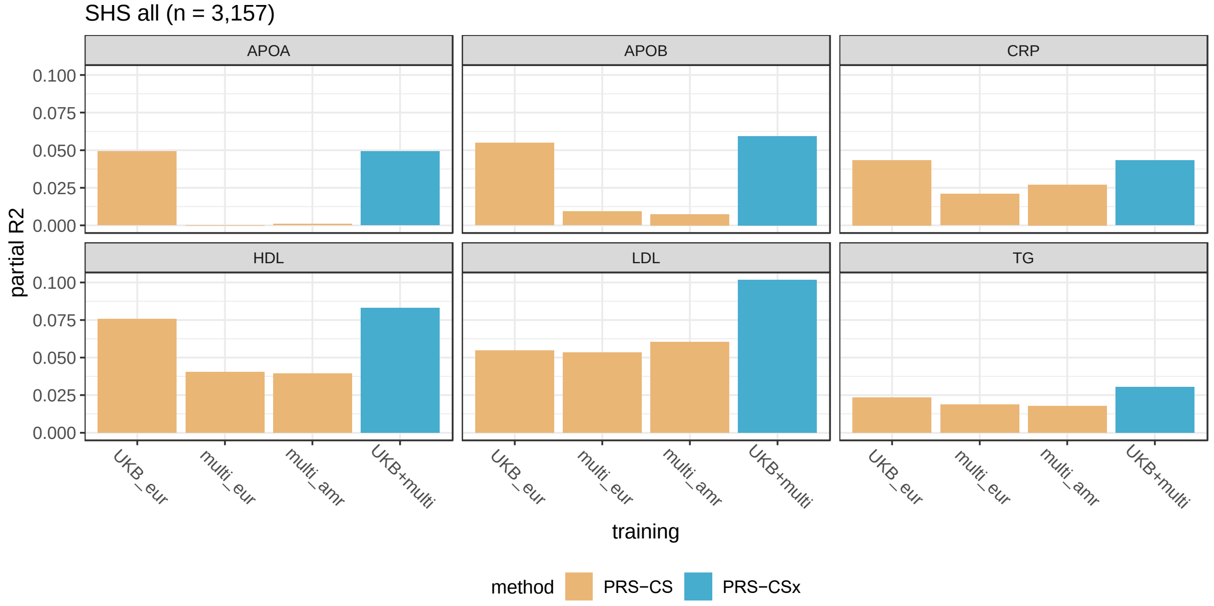

eFigure 8. Smoothscatter plots comparing the allele frequency between SHS and European population in TOP-LD.

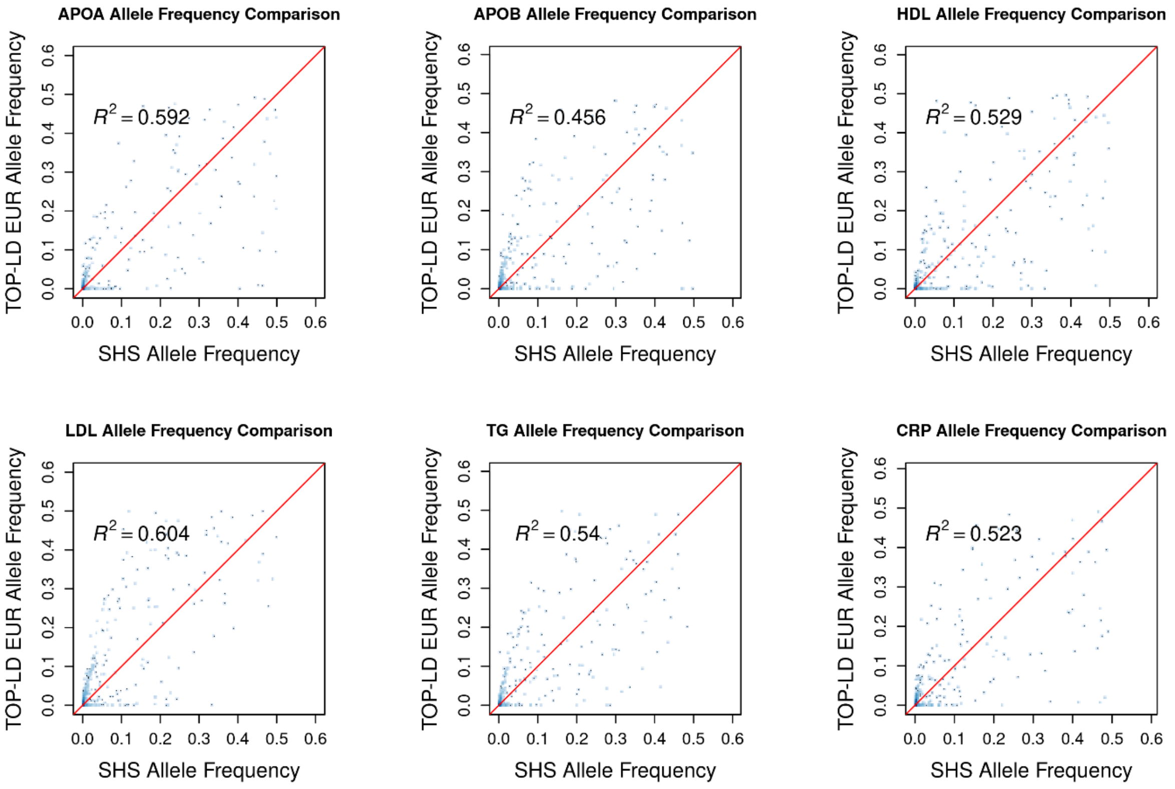

© 2025 Sun Q et al. *JAMA Network Open*.

eFigure 9. Smoothscatter plots comparing the allele frequency between SHS and African population in TOP-LD.

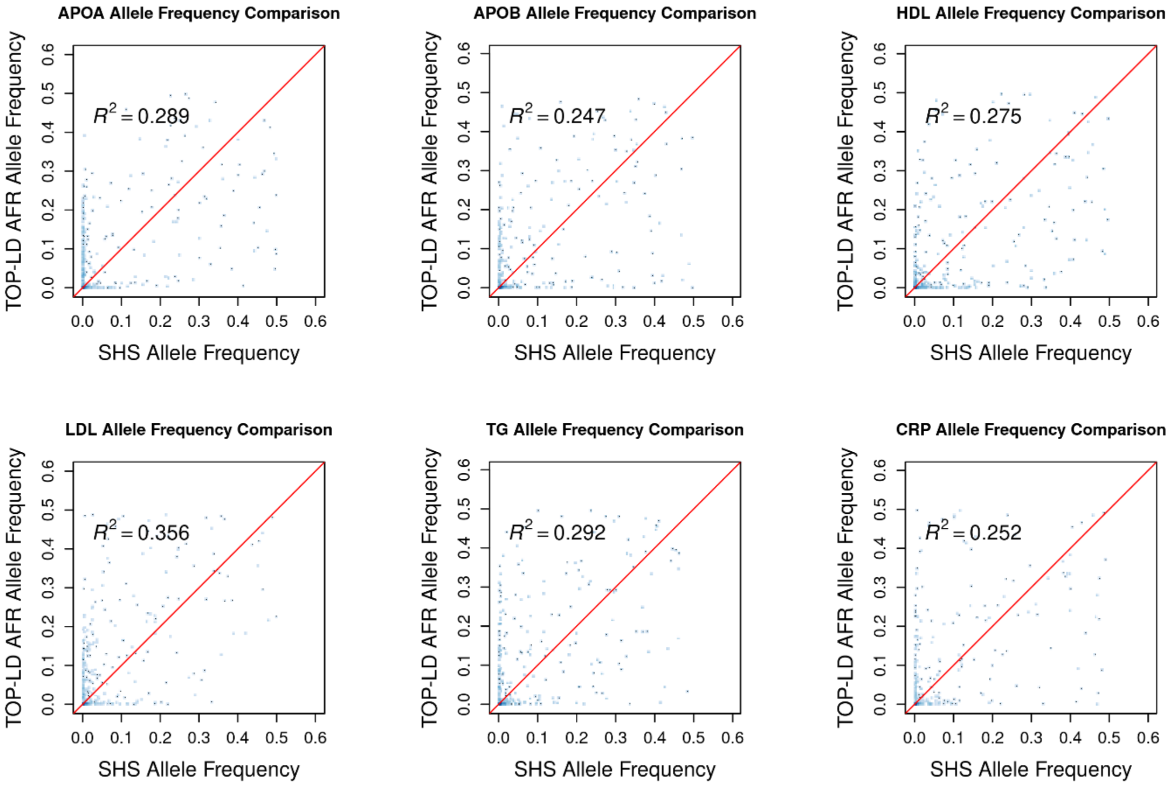

© 2025 Sun Q et al. *JAMA Network Open*.

eFigure 10. Smoothscatter plots comparing the allele frequency between SHS and East Asian population in TOP-LD.

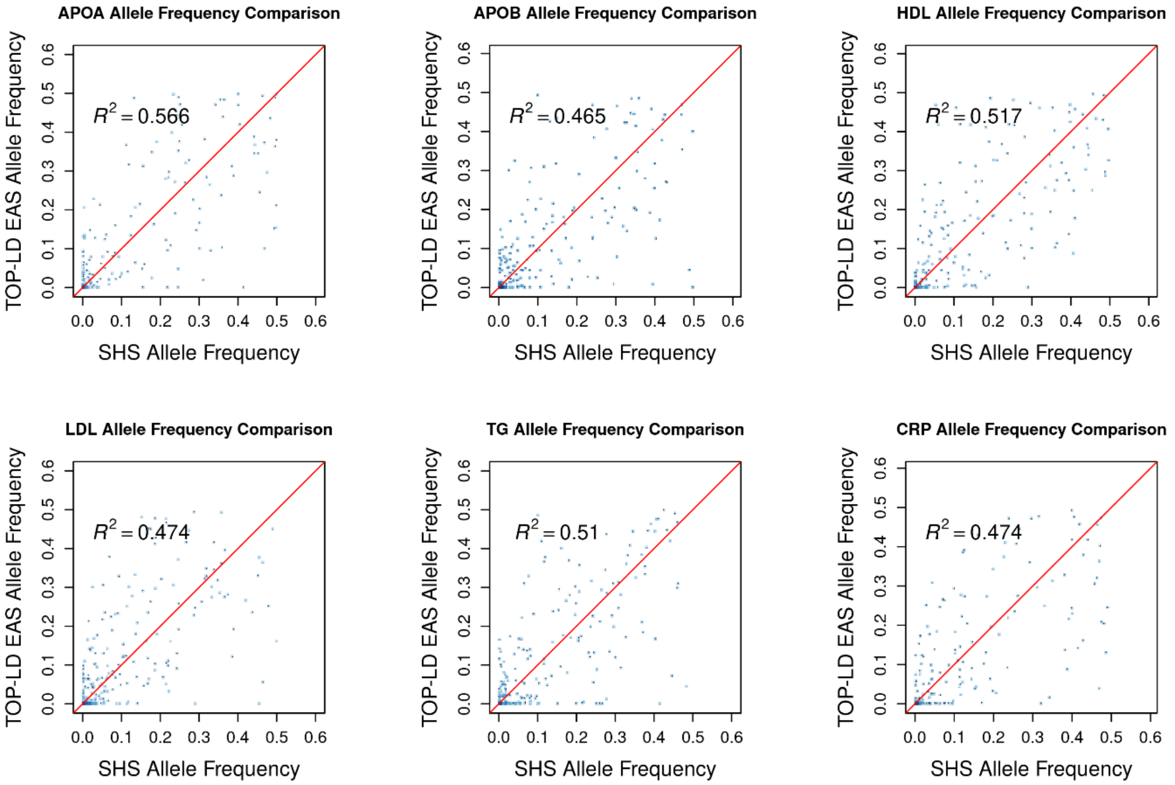

© 2025 Sun Q et al. *JAMA Network Open*.

eFigure 11. Smoothscatter plots comparing the allele frequency between SHS and South Asian population in TOP-LD.

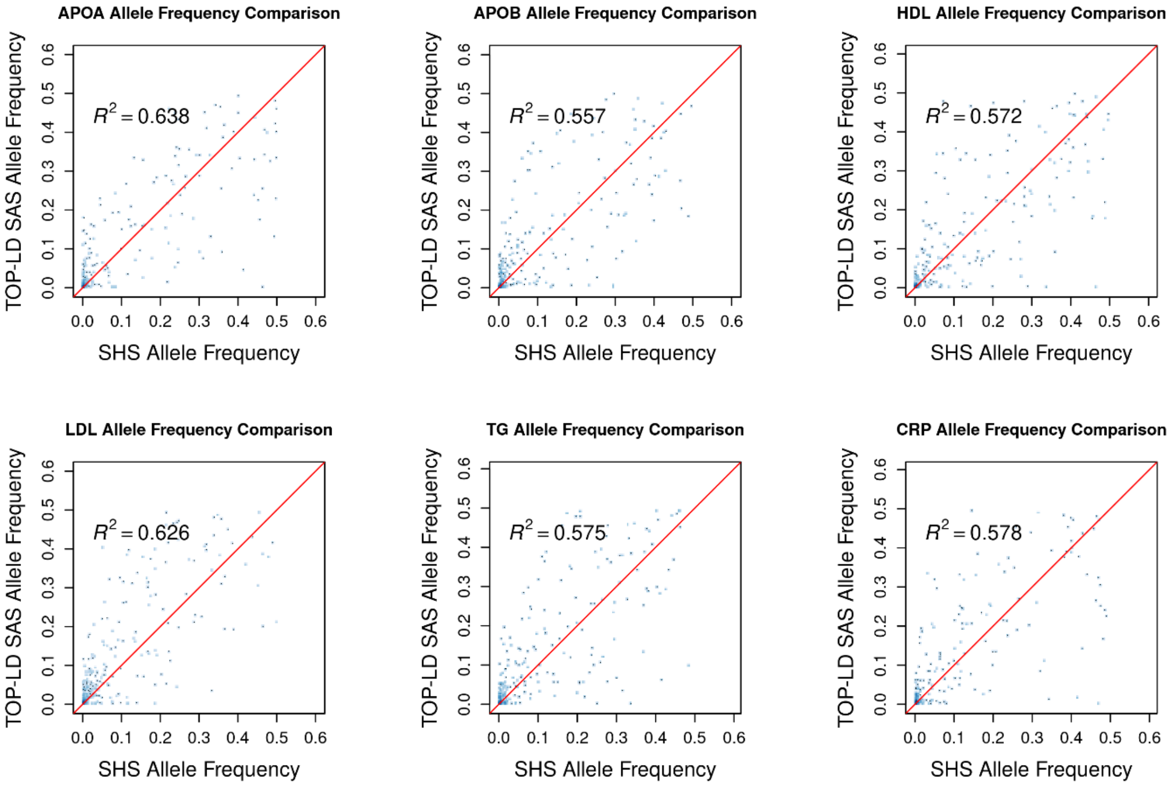

© 2025 Sun Q et al. *JAMA Network Open*.

eReferences

1. Macy EM, Hayes TE, Tracy RP. Variability in the measurement of C-reactive protein in healthy subjects: implications for reference intervals and epidemiological applications. *Clin Chem*. 1997;43(1):52-58. doi:10.1093/clinchem/43.1.52

2. Rifai N, Tracy RP, Ridker PM. Clinical efficacy of an automated high-sensitivity C-reactive protein assay. *Clin Chem*. 1999;45(12):2136-2141.

3. Robbins DC, Welty TK, Wang WY, Lee ET, Howard BV. Plasma lipids and lipoprotein concentrations among American Indians: comparison with the US population. *Curr Opin Lipidol*. 1996;7(4):188-195. doi:10.1097/00041433-199608000-00003

4. Purcell S, Neale B, Todd-Brown K, et al. PLINK: a tool set for whole-genome association and population-based linkage analyses. *Am J Hum Genet*. 2007;81(3):559-575. doi:10.1086/519795

5. Sun Q, Liu W, Rosen JD, et al. Leveraging TOPMed imputation server and constructing a cohort-specific imputation reference panel to enhance genotype imputation among cystic fibrosis patients. *HGG Adv*. 2022;3(2):100090. doi:10.1016/j.xhgg.2022.100090

6. Sun Q, Yang Y, Rosen JD, et al. MagicalRsq: Machine-learning-based genotype imputation quality calibration. *Am J Hum Genet*. 2022;109(11):1986-1997. doi:10.1016/j.ajhg.2022.09.009

7. Sun Q, Yang Y, Rosen JD, et al. MagicalRsq-X: A cross-cohort transferable genotype imputation quality metric. *Am J Hum Genet*. 2024;111(5):990-995. doi:10.1016/j.ajhg.2024.04.001

8. Taliun D, Harris DN, Kessler MD, et al. Sequencing of 53,831 diverse genomes from the NHLBI TOPMed Program. *Nature*. 2021;590(7845):290-299. doi:10.1038/s41586-021-03205-y

9. Das S, Forer L, Schönherr S, et al. Next-generation genotype imputation service and methods. *Nat Genet*. 2016;48(10):1284-1287. doi:10.1038/ng.3656

10. Loh P-R, Danecek P, Palamara PF, et al. Reference-based phasing using the Haplotype Reference Consortium panel. *Nat Genet*. 2016;48(11):1443-1448. doi:10.1038/ng.3679

11. Fuchsberger C, Abecasis GR, Hinds DA. minimac2: faster genotype imputation. *Bioinformatics*. 2015;31(5):782-784. doi:10.1093/bioinformatics/btu704

12. Kang HM, Sul JH, Service SK, et al. Variance component model to account for sample structure in genome-wide association studies. *Nat Genet*. 2010;42(4):348-354. doi:10.1038/ng.548

13. Mbatchou J, Barnard L, Backman J, et al. Computationally efficient whole-genome regression for quantitative and binary traits. *Nat Genet*. 2021;53(7):1097-1103. doi:10.1038/s41588-021-00870-7

© 2025 Sun Q et al. *JAMA Network Open*.

14. Sun Q, Graff M, Rowland B, et al. Analyses of biomarker traits in diverse UK biobank participants identify associations missed by European-centric analysis strategies. *J Hum Genet*. 2022;67(2):87-93. doi:10.1038/s10038-021-00968-0

15. Sun Q, Rowland BT, Chen J, et al. Improving polygenic risk prediction in admixed populations by explicitly modeling ancestral-differential effects via GAUDI. *Nat Commun*. 2024;15(1):1016. doi:10.1038/s41467-024-45135-z

16. Willer CJ, Li Y, Abecasis GR. METAL: fast and efficient meta-analysis of genomewide association scans. *Bioinformatics*. 2010;26(17):2190-2191. doi:10.1093/bioinformatics/btq340

17. Huang L, Rosen JD, Sun Q, et al. TOP-LD: A tool to explore linkage disequilibrium with TOPMed whole-genome sequence data. *Am J Hum Genet*. 2022;109(6):1175-1181. doi:10.1016/j.ajhg.2022.04.006

18. Ge T, Chen C-Y, Ni Y, Feng Y-CA, Smoller JW. Polygenic prediction via Bayesian regression and continuous shrinkage priors. *Nat Commun*. 2019;10(1):1776. doi:10.1038/s41467-019-09718-5

19. Ruan Y, Lin Y-F, Feng Y-CA, et al. Improving polygenic prediction in ancestrally diverse populations. *Nat Genet*. 2022;54(5):573-580. doi:10.1038/s41588-022-01054-7

20. Wojcik GL, Graff M, Nishimura KK, et al. Genetic analyses of diverse populations improves discovery for complex traits. *Nature*. 2019;570(7762):514-518. doi:10.1038/s41586-019-1310-4

21. Dai J, Niu X, Bullock A, Manson SM, O’Connell J, Jiang L. Prevalence of Diagnosed Type 1 and Type 2 Diabetes Among American Indian and Alaska Native Peoples in 2012-2013. *Diabetes Care*. 2024;47(1):e1-e3. doi:10.2337/dc23-0930

22. Howard BV, Lee ET, Cowan LD, et al. Rising tide of cardiovascular disease in American Indians. The Strong Heart Study. *Circulation*. 1999;99(18):2389-2395. doi:10.1161/01.cir.99.18.2389

© 2025 Sun Q et al. *JAMA Network Open*.
