# Supplementary material for: Polygenic Scores of Cardiometabolic Risk Factors in American Indian Adults
Source: JAMA Netw Open. 2025 Mar 12;8(3):e250535. doi: 10.1001/jamanetworkopen.2025.0535 (PMC11904716; doi:10.1001/jamanetworkopen.2025.0535)
Supplement: Supplement 3. — Data Sharing Statement [file jamanetwopen-e250535-s003.pdf]

Data Sharing Statement

Sun. Polygenic Scores of Cardiometabolic Risk Factors in American Indian Adults. *JAMA Netw Open*. Published March 12, 2025. doi:10.1001/jamanetworkopen.2025.0535

Data
Data available: Yes
Data types: Data dictionary
How to access data: The data is at: https://strongheartstudy.org. Requests will be reviewed by tribal research partners before data may be released. This policy is consistent with the NIH Policy for Data Management and Sharing: Responsible Management and Sharing of American Indian/Alaska Native Participant Data: https://grants.nih.gov/grants/guide/notice-files/NOT-OD-22-214.html#:~:text=NIH%20recognizes%20that%20conducting%20biomedical
When available: With publication

Supporting Documents
Document types: None

Additional Information

Mechanisms of data availability: The PRS catalog provides support for the datasets
Any additional restrictions: The genotype data is not publicly available due to restrictions related to consent.
